# System-level and patient-level explanations for non-attendance at diabetic retinopathy screening in Sutton and Merton (London, UK): a qualitative analysis of a service evaluation

**DOI:** 10.1136/bmjopen-2015-010952

**Published:** 2016-05-18

**Authors:** R Strutton, A Du Chemin, I M Stratton, A S Forster

**Affiliations:** 1 Sutton and Merton Diabetic Eye Screening Programme, London, UK; 2 NHS England (London), London, UK; 3 Sutton and Merton Diabetic Eye Screening Programme, London, UK; 4 Gloucestershire Retinal Research Group, Gloucester Hospitals NHS Foundation Trust, Cheltenham, UK; 5 Health Behaviour Research Centre, London, UK; 6 Department of Primary Care and Public Health Sciences, King's College London, London, UK

**Keywords:** QUALITATIVE RESEARCH, utilization of health care, DIABETES & ENDOCRINOLOGY

## Abstract

**Objectives:**

Non-attendance at diabetic retinopathy screening has financial implications for screening programmes and potential clinical costs to patients. We sought to identify explanations for why patients had never attended a screening appointment (never attendance) in one programme.

**Design:**

Qualitative analysis of a service evaluation.

**Setting:**

One South London (UK) diabetic eye screening programme.

**Participants and procedure:**

Patients who had been registered with one screening programme for at least 18 months and who had never attended screening within the programme were contacted by telephone to ascertain why this was the case. Patients’ general practices were also contacted for information about why each patient may not have attended. Framework analysis was used to interpret responses.

**Results:**

Of the 296 patients, 38 were not eligible for screening and of the 258 eligible patients, 159 were not contactable (31 of these had phone numbers that were not in use). We obtained reasons from patients/general practices/clinical notes for non-attendance for 146 (57%) patients. A number of patient-level and system-level factors were given to explain non-attendance. Patient-level factors included having other commitments, being anxious about screening, not engaging with any diabetes care and being misinformed about screening. System-level factors included miscommunication about where the patient lives, their clinical situation and practical problems that could have been overcome had their existence been shared between programmes.

**Conclusions:**

This service evaluation provides unique insight into the patient-level and system-level reasons for never attendance at diabetic retinopathy screening. Improved sharing of relevant information between providers has the potential to facilitate increased uptake of screening. Greater awareness of patient-level barriers may help providers offer a more accessible service.

**Strengths and limitations of this study**

- Participants were never attenders at one diabetes eye screening programme, who are a rarely researched population, as by definition they do not engage with diabetes eye screening.
- Significant effort was made to contact all never attenders; there was no limit to the number of contacts attempted until a patient was reached.
- Patients’ general practices (GPs) were also contacted to ascertain why a patient may not have attended diabetes eye screening, offering a triangulation of findings.
- We ascertained reasons for non-attendance for only 57% of patients; reasons for non-attendance among patients who were not contactable may differ from those who were.
- Responses were recorded as detailed notes by the member of screening staff who contacted the patients and providers; however, responses may not have been recorded verbatim.

## Introduction

Almost four million individuals in the UK have been diagnosed with diabetes mellitus^1^ and an estimated 347 million worldwide.^2^ Diabetic retinopathy and maculopathy are complications of diabetes mellitus that can affect vision. Estimates suggest that around 5% of blindness globally is caused by diabetic retinopathy and this is higher in Western European countries (17%).^3^ Diabetic eye disease can be detected early and treatments are available to prevent blindness if given at an early stage of the disease.^4^ ^5^ Organised diabetic eye screening programmes exist in a number of Western European countries, including in England. Individuals who have diabetes mellitus, who are over the age of 12 are invited to attend screening every year through a call and recall programme. There is variation across the UK in how screening is delivered: some programmes have dedicated hospital clinics, whereas others are based in GP surgeries or high-street optometrists. Patients who have sight threatening diabetic retinopathy (STDR) detected (defined as moderate or severe non-proliferative retinopathy or proliferative retinopathy or referable maculopathy) are referred for treatment or more frequent monitoring.

Non-attendance at diabetic eye screening is costly to the UK National Health Service (NHS), with one programme estimating that non-attendance cost >£78 000 ($125 000 or €97 000) over 1 year.^6^ In quarter 3 of 2013/2014 around 83% of patients invited for diabetic eye screening took up the offer.^7^ Many of the 17% who were not screened will have attended in previous years, but a proportion of patients have never attended for screening despite being eligible. Patients who do not attend for diabetic eye screening have risk factors that make them more vulnerable to diabetic retinopathy, including having poorer A1C and blood pressure control and are more likely to have been diagnosed with diabetes for longer.^8^ ^9^ Patients who do not attend for screening frequently are at increased risk of STDR and risk increases with the duration that an individual is unscreened.^10^

Patients who do not attend for screening are a vulnerable population and some research has sought to consider the reasons why patients may not be attending for diabetic retinopathy screening. Barriers reported have included patients believing that they do not have diabetic retinopathy, embarrassment about poor glycaemic control, anxiety about treatment, conflicting priorities, believing that other hospital eye appointments or regular optometrist appointments test for diabetic retinopathy and lack of awareness of the importance of screening.^11–14^ Service-level barriers have only been considered in research that has been conducted in the context of screening performed at GP surgeries or high-street optometrists and not community health clinic-based screening.^15^ In addition, much of this work was conducted outside of the UK, so may not be reflective of the UK context, where screening is offered free-at-the-point-of-receipt through an organised call and recall system. Research that has considered service-level barriers has suggested that we need a greater understanding of the communication between retinopathy screening stakeholders (ie, general practice and screening programmes).^15^

We report the findings of a service evaluation of all active patients who were registered with one diabetic eye screening programme for at least 18 months and who had never attended screening at one community health clinic screening programme (n=296). The study sought to explore the patient-level (ie, those determined to some extent by the patient) and system-level (ie, those determined by the healthcare provider) reasons for never attendance at diabetic eye screening.

## Methods

The service evaluation was based in one South London diabetic eye screening programme (there are 61 such programmes in the UK). In this programme screening is organised centrally and appointments offered in dedicated community health clinics. In 2012/2013 this screening programme had 18 334 registered patients on their database, and an uptake rate of 84%. The screening programme identified all active patients, registered on the screening database for at least 18 months, who had never attended diabetic eye screening within the programme as of 31 March 2012 (n=296). Patients and general practice staff were contacted by a female member of the screening programme staff (RS) to ascertain why patients had not attended for screening. General practice staff were GPs, nurses and administrative staff. RS was qualified to make such enquiries as it was part of her usual job, but this means that she was also interested in increasing attendance. As patients were never attendees at this service, RS did not have a relationship with patients prior to the study. Detailed notes were recorded for each contact made with patients and providers. Existing clinical notes on primary care and community databases were also searched systematically. A review of consent and eligibility coding on primary care databases was undertaken and this information was cross-referenced with patients’ status on the screening database to indicate where further relevant information may be held by primary care. Reasons given to explain why patients wanted to opt out of the programme during the period were recorded for those who returned opt-out forms. Three standardised emails were sent to non-responding practices and each practice received two phone calls which were initiated in the same way. The member of screening staff attempted to contact eligible patients between 1 October 2012 and 31 March 2013, including on weekends (there was no limit to the number of contacts attempted; mean number of contacts=2.5 per patient). Patient phone conversations were initiated in the same way, but conversations inevitably differed beyond that. Patients’ age, gender and ethnicity were collected where available. The same number of data sources was accessed for each patient; there was no hierarchy (ie, if contact was made with a patient, their provider was still contacted and clinical notes still searched).

Framework analysis was used to qualitatively organise the patients’ and providers’ responses and identify categories that arose from the data.^16^ This flexible method of coding allows easy retrieval of information within and between cases. The process of charting is transparent allowing others to judge the reliability of the interpretation of the data. Analysis comprises five stages: familiarisation, the development of a thematic framework, indexing, charting and mapping. Patients’ and providers’ responses were coded using the thematic framework in Microsoft Excel. A second rater reviewed 10% of cases. Agreement between raters was moderate (Cohen's κ=0.71, p<0.001). Disagreements were resolved by discussion. Quotes are provided to give examples of the content of the codes. The quotes are taken from the detailed notes of telephone/email contact with the patient or provider or as recorded in clinical notes.

These data were collected as part of a service evaluation (CQUIN) and performed in line with the provider's Trust guidelines.

## Results

### Descriptive characteristics of the sample

There were 296 active patients registered on the screening database for at least 18 months who had never attended diabetic eye screening within the programme as of 31 March 2012 (at this one screening programme). Contact with patients and providers during the study identified 38 patients who were ineligible for screening, including patients who were not diabetic, who had attended previously and so were not due to be screened, who were under ophthalmology care, who were deceased and who were being seen at another eye screening programme. Of the remaining 258 patients, practices provided reasons for non-attendance for 90 patients. During the period, 159 patients were not contactable (31 of these had telephone numbers that were not in use/had no contact number) and 21 patients did not give a meaningful reason for their non-attendance. We obtained reasons from patients, providers or clinical notes for 146 (57%) patients.

The gender split was balanced in the sample (53% men). The greatest proportion of patients was in the age group 54–74 years (40%; table 1). Patients’ ethnicity and main language spoken was available for 162 (62%) and 78 (30%) patients, respectively. Where known, most patients were from a White ethnic background (36%), followed by patients from an Asian or Asian British background (19%). Of patients whose first language was known, most spoke English (81%). There were 35 patients who were identified as having no general practitioner (14%; table 1). During the study, 15 patients opted out of the screening programme (6%) by returning a signed opt-out form in line with national policy.

**Table 1:** Patients’ demographic characteristics (n=258)

	n	Per cent
Ethnicity		
White	94	36.4
Black or Black British	15	5.8
Asian or Asian British	49	19.0
Mixed	4	1.6
Unknown	96	37.2
Main language spoken		
English	63	24.4
Other	15	5.8
Unknown	180	69.8
Age (group in years)		
12–34	33	12.8
35–54	80	31.0
55–74	103	39.9
≥75	41	15.9
Missing	1	0.4
No general practitioner	35	13.6

### Qualitative analysis

A number of patient-level and system-level factors were given to explain non-attendance.

#### Patient-level factors

##### Other commitments

For a number of patients, competing factors were prioritised more than screening, including having work and childcare commitments, personal or family illness and bereavements. Others were out of the area or country for a period of time and so could not attend. Such reasons were reported by patients and providers.

I [screening staff] called patient and his mother said that he is very ill at the moment…She said that he vomits a lot so it is difficult to get to appointments. Male, 35 to 54 years.

Patient … said it's difficult to attend because her daughter has had a break down and she is looking after her children. Female, 55 to 74 years.

Patient called to apologise for missing his appointment this morning as he is … on business at present. He said he was aware he has missed a few now and needs to be seen. Male, 35 to 54 years.

##### Anxiety about screening

The most common anxiety expressed was that of patients disliking the eye drops used during screening (some anticipated disliking them, whereas others may have received drops if screened by another programme previously). This was reported by patients and providers. Other patients expressed a preference to have a family member accompany them, and it was their family member's unavailability that had prevented them from attending (in spite of transport being offered by the screening programme).

Patient apologised for causing any problems and said she had the drops once and remembers it was ok but has since read that they can sting and she has built up a phobia about it. Female, 35 to 54 years.

##### Disengagement with diabetes care in general

Some relatives of patients and general practice staff reported that patients had disengaged with their diabetes care in general.

[Patient] is refusing to even discuss his condition, so all you can do is keep sending invites. Male, 55 to 74 years.

[Father] told me [screening staff] that she [patient] refuses to go to any appointments even though both he and her mother encourage her. He said that the diabetes nurse told them she is in denial about her diabetes and that she has been this way since she was diagnosed. Female, 12 to 34.

##### Misinformed about screening

A number of patients and a few general practice staff provided reasons for their/their patients’ non-attendance that demonstrated them being misinformed. These included: not understanding that diabetic retinopathy screening is not performed as part of a standard optician eye test, not knowing where the screening clinic is, patients believing that they are not diabetic (although confirmed by GP) and patients being seen in a glaucoma clinic and perceiving this to be sufficient diabetes eye care.

Patient said … her opticians do all her eye checks. Female, 35 to 54 years.

##### Forgetting

One patient reported that their non-attendance at screening was due to them forgetting to attend.

#### System-level factors

There were also a number of system-level factors that were related to screening non-attendance. Many of these could have been overcome had there been better communication between services.

##### Miscommunication about patients’ residence

Some patients were reported by general practice staff to be known to be out of the area/country, some permanently. A small number of patients were known by general practice staff to have no fixed abode.

##### Practical problems

There were also practical problems that were barriers to screening attendance among patients, but could have been overcome had the screening programme been aware of them. These included patients being housebound (reported by providers) and having transport problems (transport problems included general practice staff not knowing how to book patient transport; reported by patients and providers).

[Patient] called the GP to say that his transport has not turned up. I [screening staff] called… transport and they said that they went to an address but there was no answer (this was his old address). I advised that this is not his address…He apologised and said he will call the patient and get him here today. Male, 55 to 74 years.

##### Invitation letter not received/not received in time

For some patients, issues with their post made it difficult for them to attend. It is important to note that appointment letters are sent out 3–4 weeks in advance of appointments.

[Patient] said that normally when she receives our letters it is the day before the appointment so there is not enough notice. She believes there may be a problem with her post. Female, 35 to 54 years.

[Patient] says he does not receive our letters because someone where he lives throws them away. He asked for them to be sent to his work. Male, 35 to 54 years.

#### Clinical notes not being shared

Eligibility and consent codes applied to patients’ primary care records were cross-referenced with patients’ statuses on the screening database. This identified one patient whom their GP had recorded ‘not indicated for diabetic retinopathy screening’, one patient who was coded as being unsuitable for digital retinal photography, 31 patients whom their GP had recorded as having ‘refused diabetic retinopathy screening’ and 40 patients who were ‘exempted from diabetes quality indicators’ in the practice. All of these patients were recorded as eligible for screening on the screening database system.

## Discussion

This service evaluation sought to explore patient-level and system-level explanations for never attendance at diabetic eye screening at this programme. Patient-factors identified during the service evaluation included having other commitments, being anxious about screening, patients not being engaged with any of their diabetes care and being misinformed about screening. System-level factors included miscommunication about patients’ residences and practical problems that could have been resolved if they were communicated between service providers.

Many of the patient-level barriers to diabetic eye screening have been reported previously,^11–13^ however, the system-level factors rarely come out of the published literature and our study provides new knowledge in this area.^14^ ^15^ Where they have been considered, communication issues between GPs and Diabetes Eye Screening Programmes have previously been reported in the context of GP surgery-based services.^15^ Uniquely, in our service evaluation, a number of patients were found to be either temporarily or permanently ineligible for screening. Many of the telephone numbers available for patients were no longer in use, suggesting that this population is highly mobile. Better communication between GP surgeries and screening programmes involving more streamlined methods of transferring relevant information will help ensure that screening lists only include eligible individuals. It may be useful for commissioners and general practices to review the systems currently in place to communicate this information to the screening programmes, to make sure that it is intuitive and simple for practices to do. Screening providers are currently penalised for the non-attendance of patients who are actually ineligible for screening. Better communication could also facilitate patients who require transport to attend screening. It would also be useful for general practices to inform screening programmes if they know that their patient will be out of the country for a period of time. Previous research has also raised the concern that patients being abroad for periods of time means that they miss the annual screening cycle.^15^ Some patients were known to have no fixed abode; these patients are a vulnerable group and screening programmes and GPs need to find ways to support their attending screening. Some of the patient-level factors influencing screening attendance will be more difficult for screening programmes to surmount, particularly personal or family illness and bereavement. Currently diabetes eye screening programmes continue to invite patients regardless of non-attendance (unless an opt-out form is returned) and such a strategy may be beneficial to patients with temporary personal issues, as the findings of this study suggest that they will attend when they are able to. Patients’ work commitments being a barrier to screening attendance could be overcome by increased awareness of extended opening hours. Some of the non-attendees were misinformed or anxious about screening. Screening programmes may be able to improve their invitation letter or information materials so that anxiety is better managed. It might be useful to review existing patient information with a group of patients to ensure that directions to the screening clinics and accessibility information are easily understood.

A number of patients were considered to be disengaged with all of their diabetes care, and as such represent a vulnerable group of patients as it is known that non-attenders are more likely to have poorer A1C and blood pressure control and are more likely to have been diagnosed with diabetes for longer.^8^ ^9^ These ‘hard to reach’ groups have been described previously.^15^ Disengaged and very resistant patients are a difficult group for screening programmes to engage, and it might be easier or more appropriate for the GP or practice nurse to persist in encouraging these patients to participate in their diabetes care, while recognising that it is patients’ choice and responsibility to look after their health.

There are some notable limitations to this service evaluation. We were only able to ascertain reasons for non-attendance for 57% of eligible patients. Non-attenders are a notoriously difficult population to conduct research with and our findings provide insight into the reasons for their non-attendance. However, the reasons for non-attendance among patients who were not contactable may differ from those who were. Responses were recorded as detailed notes by the member of screening staff who contacted the patients and providers; however, there remains the possibility that responses were not recorded verbatim. The reasons that patients’ provided may have been subject to responder bias, whereby they gave answers that they thought the screening programme wanted to hear. Similarly, clinical notes and general practice staff perceptions’ may not accurately reflect patients’ reasons for non-attendance. It would have been interesting to explore associations between patients’ reasons for non-attendance and their diabetes care or control; however, the UK diabetes eye screening programmes do not routinely have access to clinical data. All patients spoken to during the service evaluation could speak English (although some had limited English). The information provided to patients about the screening programme is available in languages other than English, but this is not made clear on the standard information. Previous research has indicated that language barriers affect attendance,^15^ but we were unable to explore this in our study. While patients were considered to be non-attenders at this one screening programme, it is possible that they had attended screening at least 18 months previously at another screening programme. Our findings may not be reflective of patients registered with other screening programmes. Finally, while our results are likely to be generalisable to other programmes in the England that have similar populations, the system factors may differ between programmes that employ different screening models. Our results may also not be generalisable to programmes in other countries that employ both different models of screening and have a different screening population.

## Conclusions

Improved sharing of relevant information between healthcare services has the potential to facilitate increased uptake of diabetic eye screening in patients who have not previously attended screening. Increased awareness of patient-level barriers may be used by screening programmes to provide a more accessible service.

## References

[1] Diabetes. Facts and stats. http://www.diabetes.org.uk/Documents/Position20statements/Facts20and20stats20June%202015.pdf (accessed 15 Mar 2016).

[2] Danaei G, Finucane MM, Lu Y National, regional, and global trends in fasting plasma glucose and diabetes prevalence since 1980: systematic analysis of health examination surveys and epidemiological studies with 370 country-years and 2.7 million participants. Lancet 2011;378:31–40. 10.1016/S0140-6736(11)60679-X 21705069

[3] Resnikoff S, Pascolini D, Etya'ale D Global data on visual impairment in the year 2002. Bull World Health Organ 2004;82:844–51. 15640920 PMC2623053

[4] Early Treatment Diabetic Retinopathy Study research group. Photocoagulation for diabetic macular edema. Early Treatment Diabetic Retinopathy Study report number 1. Early Treatment Diabetic Retinopathy Study research group. Arch Ophthalmol 1985;103:1796–806. 2866759

[5] Diabetic Retinopathy Study research group. Photocoagulation treatment of proliferative diabetic retinopathy: relationship of adverse treatment effects to retinopathy severity. Diabetic retinopathy study report no. 5. Dev Ophthalmol 1981;2:248–61. 7262408

[6] Waqar S, Bullen G, Chant S Cost implications, deprivation and geodemographic segmentation analysis of non-attenders (DNA) in an established diabetic retinopathy screening programme. Diabetes Metab Syndr 2012;6:199–202. 10.1016/j.dsx.2012.08.009 23199538

[7] NHS screening programmes. KPI reports 2014 to 2015. https://www.gov.uk/government/publications/nhs-screening-programmes-kpi-reports-2014-to-2015 (accessed 15 Mar 2016).

[8] Gulliford MC, Dodhia H, Chamley M Socio-economic and ethnic inequalities in diabetes retinal screening. Diabet Med 2010;27:282–8. 10.1111/j.1464-5491.2010.02946.x 20536490

[9] Leese GP, Boyle P, Feng Z Screening uptake in a well-established diabetic retinopathy screening program: the role of geographical access and deprivation. Diabetes Care 2008;31:2131–5. 10.2337/dc08-1098 18728235 PMC2571062

[10] Forster AS, Forbes A, Dodhia H Non-attendance at diabetic eye screening and risk of sight-threatening diabetic retinopathy: a population-based cohort study. Diabetologia 2013;56:2187–93. 10.1007/s00125-013-2975-0 23793717

[11] Lewis K, Patel D, Yorston D A qualitative study in the United Kingdom of factors influencing attendance by patients with diabetes at ophthalmic outpatient clinics. Ophthalmic Epidemiol 2007;14:375–80. 10.1080/09286580701375195 18161611

[12] Maberley DA, Koushik A, Cruess AF Factors associated with missed eye examinations in a cohort with diabetes. Can J Pub Health 2002;93:229–32. 12050993 10.1007/BF03405006 PMC6979876

[13] Walker EA, Basch CE, Howard CJ Incentives and barriers to retinopathy screening among African-Americans with diabetes. J Diabetes Complicat 1997;11:298–306. 10.1016/S1056-8727(96)00121-3 9424171

[14] Sachdeva A, Stratton IM, Unwin J Diabetic retinopathy screening: study to determine risk factors for non-attendance. Diabetes Prim Care 2012;14:308–16.

[15] Lindenmeyer A, Sturt JA, Hipwell A Influence of primary care practices on patients’ uptake of diabetic retinopathy screening: a qualitative case study. Brit J Gen Pract 2014;64:e484–92. 10.3399/bjgp14X680965 25071061 PMC4111341

[16] Spencer L, Ritchie J, O'Connor AM Analysis: practices, principles and processes. In: Ritchie J, Lewis J, eds. Qualitative research practice: a guide for social science students and researchers. London: SAGE Publications, 2003:199–218.

